# Changing Trends of Odontogenic Cysts and Tumors in Kenya: A 20-Year Retrospective Analysis

**DOI:** 10.7759/cureus.70471

**Published:** 2024-09-29

**Authors:** Joseph Mutio, Elizabeth Dimba, Krishan Sarna, Khushboo Sonigra, Wambeti Twahir, Kanini Ndui, Ochiba O Lukandu, Walter Odhiambo, Wei Cheong Ngeow

**Affiliations:** 1 Department of Oral and Maxillofacial Surgery, Oral Pathology, and Oral Medicine, Division of Oral Pathology, University of Nairobi, Nairobi, KEN; 2 Department of Oral and Maxillofacial Surgery, Oral Pathology, and Oral Medicine, Division of Oral and Maxillofacial Surgery, University of Nairobi, Nairobi, KEN; 3 Department of Oral Pathology, Kenyatta National Hospital, Nairobi, KEN; 4 Department of Maxillofacial Surgery, Oral Medicine and Oral Pathology, Moi University, Eldoret, KEN; 5 Department of Oral and Maxillofacial Clinical Sciences, Faculty of Dentistry, Universiti Malaya, Kuala Lumpur, MYS

**Keywords:** adenomatoid odontogenic tumor, dentigerous cyst (dc), odontogenic ameloblastoma, odontogenic cysts and tumours, oral and maxillofacial pathology

## Abstract

Objectives: This study endeavors to bridge the long-term diagnostic and management gap through a comprehensive audit of odontogenic cysts and tumors in Kenya, offering crucial insights for both clinicians and policymakers.

Methods: Patient records (2001-2020) with odontogenic cysts and tumors were retrospectively abstracted from two major referral hospitals in Nairobi, Kenya, covering demographics, lesion location, and histological diagnosis. IBM SPSS Statistics for Windows, Version 29.0 (Released 2023; IBM Corp., Armonk, New York, United States) was used for data analysis which included all descriptive statistics, student t-tests, chi-square tests, and one-way ANOVA.

Results: A total of 1889 cases were analyzed. Males exhibited a significantly higher prevalence of cysts and tumors (p < 0.001). Odontogenic tumors dominated in the age group of 21-30 years, while cysts were prevalent in the age group of 11-20 years. Ameloblastoma was the most prevalent tumor (n=853; 84.1%) while dentigerous cysts were the most common cysts (n = 468; 53.5%), both demonstrating a male predilection. The mean age at presentation and site predilection of each tumor and cyst were also detailed.

Conclusion: This study provides crucial insights into odontogenic cysts and tumors in Kenya emphasizing geographic variations, age-specific patterns, and gender disparities for more effective diagnostic and management strategies, especially in resource-limited settings, to improve outcomes and reduce associated morbidity and mortality.

## Introduction

In 2004, a report described the oral health infrastructure in Kenya, together with problems associated with its health financing [1]. Among other things, it described that the incidence of oral cancer was very low at the time, accounting for 2% of all malignancies. Unfortunately, there was no mention of the prevalence of odontogenic cysts and tumors in Kenya. Although not malignant, odontogenic cysts and tumors also stand out as prevalent entities that significantly impact an individual’s well-being and the broader healthcare landscape, as has been reported in several African countries [2]. There are a handful of reports on the prevalence of odontogenic tumors in the African continent, and up to now, countries like Nigeria, Ghana, South Africa, Ethiopia, Zimbabwe, Algeria, and Egypt contributed to our current knowledge of the African scene [3-10]. Of interest is the fact that many of these publications originated from Nigeria, the authors of which also contributed a dominant body of work on odontogenic cysts. Other countries with similar types of reports were Ghana, Libya, and South Africa [2,11-13].

Reports on odontogenic cysts and tumors from Kenya on the other hand, have been limited, with the earliest report dating back to 1973 for oral tumors [14] and 1987 for jaw cysts [15]. More updated reports were published in 2007 and 2011, respectively [16,17]. In addition, one specific report looking into ameloblastoma in children was published more than 10 years ago [18]. There is, however, a vacuum throughout the last decade which was partially answered by Ogundana et al. [2]. The lack of such information makes it hard for healthcare workers to anticipate their workload and distribute resources, given the limited number of specialists in the country [1].

In the context of odontogenic pathology, a cyst is characterized as an epithelial-lined pathological cavity enveloping fluid within a capsule, while a tumor is described as a proliferating mass of cells, categorized as either benign or malignant [19]. Over time, the diagnostic criteria for the classification of odontogenic cysts and tumors have been progressively refined. Notably, the fifth edition of the WHO Classification of Head and Neck Tumors, first made available online in 2022 in a digital format, advanced the classification of odontogenic tumors and cysts of the jaw by integrating histogenesis, clinical characteristics, molecular biology, and genetics as key parameters [20,21].

In the majority of reports, ameloblastoma appears to be the most common odontogenic tumor reported in Africa, while dentigerous cysts and radicular cysts are the most common jaw cysts observed [2-4,6,8-11,13,17,22,23]. Previous findings from Kenya appeared to report the same except for the fact that odontogenic keratocyst topped the list of cystic lesions [16,17]. This difference may have resulted from the changes in classifying this lesion as a tumor in other/previous studies. The current study aims to provide an update on the prevalence of odontogenic cysts and tumors in Kenya, i.e. to determine if there are any changes since those last reports. In addition, as one report from West Africa described a relatively high incidence of ameloblastomatous change in radicular cysts, in comparison to the low prevalence of radicular cysts diagnosed, the authors would like to determine if the same has happened in the eastern part of Africa [24]. Lastly, given the low prevalence of oral cancer in Kenya, the authors wish to determine if ameloblastic carcinoma had any contribution to the number of oral cancers in this part of the world [25]. This is because ameloblastoma is the most common tumor reported in Kenya, and reports from the western part of Africa were inclined to demonstrate a substantial incidence of malignant changes [4,6,16].

## Materials and methods

**Figure 1:**
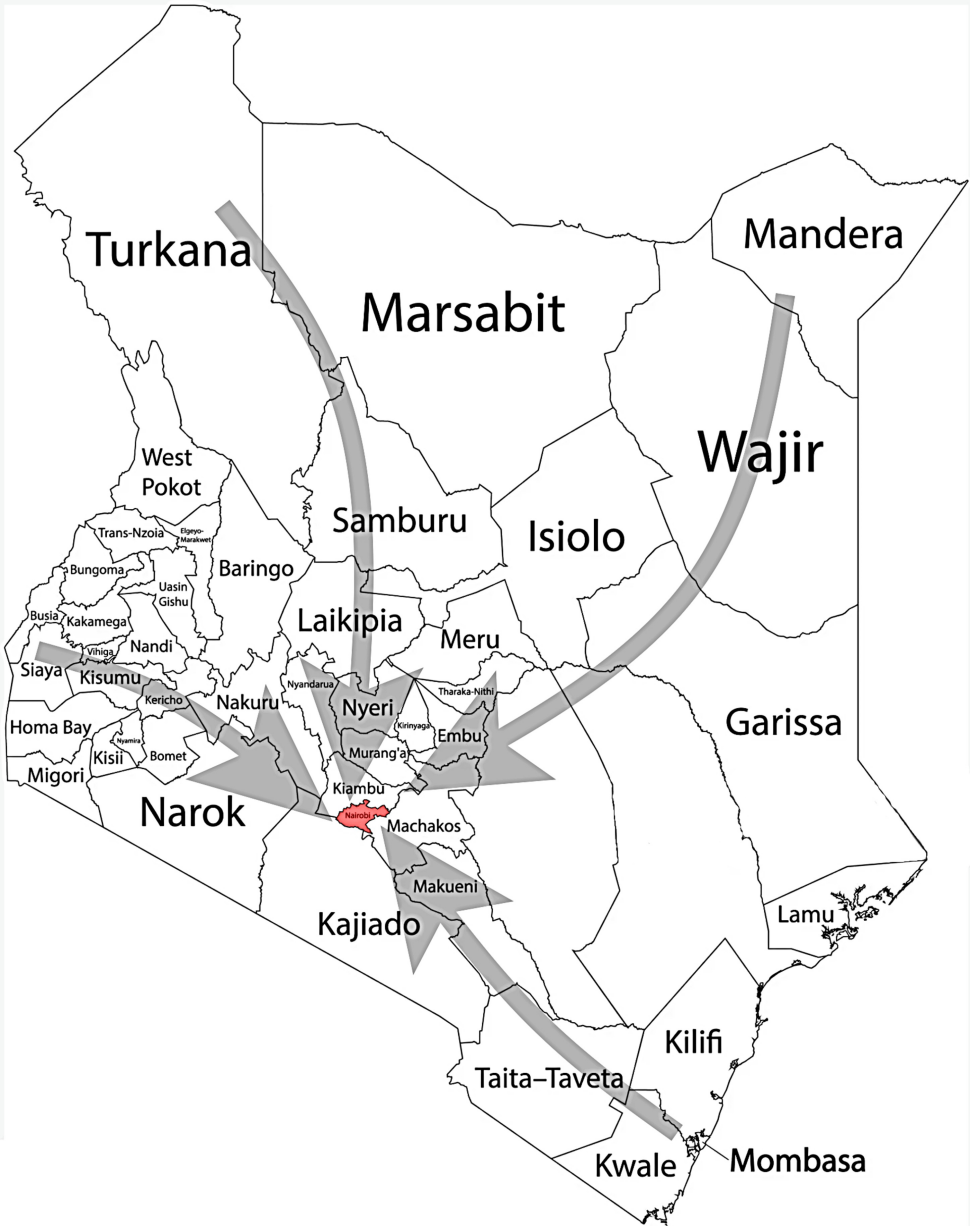
A detailed map of Kenya showing the 47 counties in the country. The grey arrows indicate that all specimens (from all 47 counties) were received in Nairobi where analysis was performed. Image Credit: Khushboo Sonigra, Krishan Sarna

A comprehensive retrospective study was conducted with records of patients spanning two decades from January 2001 to December 31, 2020, for a substantial cohort of patients diagnosed with odontogenic cysts and tumors. Data for this investigation were meticulously gathered from two prominent oral and maxillofacial referral centers in Nairobi, Kenya: the University of Nairobi Dental Hospital (UoNDH) and the Kenyatta National Hospital (KNH). These institutions, renowned for their expertise, served as the primary sources of information, ensuring the inclusion of diverse cases. These two centers receive pathology specimens from all 47 counties within Kenya, that have a combined population of 53 million (Figure 1). Approval for the study was sought and granted by the Kenyatta National Hospital-University of Nairobi Ethics & Research Committee under protocol number P224/04/2021. The research was conducted in strict compliance with the principles outlined in the World Medical Association Declaration of Helsinki. 

Over the investigation period, a detailed examination was conducted on 25,032 records derived from 15,319 specimens processed at the UoNDH and 9,713 specimens at the KNH. Within this extensive dataset, a total of 1,889 patients were identified with diagnoses of odontogenic cysts or tumors. Each patient's clinical file was reviewed and analyzed in detail for data collection encompassing variables such as age, gender, anatomical location of the lesion (maxilla, mandible, or soft tissue), and histopathological diagnosis. To ensure the robustness of the study, specific inclusion and exclusion criteria were applied. Inclusion criteria encompassed patients diagnosed with either odontogenic cysts or tumors, without age or gender restrictions. The exclusion criteria consisted of patients with incomplete clinical records, cases lacking a histopathological diagnosis, and instances classified as non-primary tumors. Patients with recurrent cysts or tumors were excluded to prevent duplicate entries over the 20-year study period, as recurrences were likely within this timeframe. Including such cases could introduce confounding effects related to prior treatments, compromising the accuracy of an epidemiological analysis focused on primary cysts and tumors. It is crucial to note that of the records identified with a diagnosis of odontogenic cysts and tumors, 17 (0.9%) were incomplete; thus, after their exclusion, the final number of cases was 1889. The condition of the dataset was therefore appropriate for such a detailed analysis. 

The histopathological diagnoses were meticulously categorized in alignment with the WHO classification of odontogenic cysts and tumors, as outlined in Tables 1-2, made available online in 2022 [20]. Notably, it is imperative to emphasize the temporal context of this investigation, spanning 20 years, within which a total of three distinct re-classifications of odontogenic cysts and tumors took place. Each of these re-classifications introduced new sub-categories for certain lesions, such as ameloblastoma. Consequently, due to the evolving nature of the classifications, earlier data records could not capture specific details of the sub-division of some lesions. Thus, in this study, the analysis of ameloblastoma was treated as a singular entity rather than being divided into various categories, recognizing the limitations imposed by the evolving diagnostic criteria over the study duration. Furthermore, some of the lesions described in the 2022 classification are known to be quite rare and indeed were not encountered during the study.

**Table 1:** The 2022 WHO classification of odontogenic tumors of the jaws

Pathology	Tumor Class	Tumor Subtype
Odontogenic Tumors	Benign Epithelial Odontogenic Tumors	Adenoid Ameloblastoma
		Adenomatoid Odontogenic Tumor
		Ameloblastoma, Conventional
		Ameloblastoma, Extraosseous/Peripheral
		Ameloblastoma, Unicystic
		Calcifying Epithelial Odontogenic Tumor
		Metastasizing Ameloblastoma
		Squamous Odontogenic Tumor
	Benign Mesenchymal Odontogenic Tumors	Cemento-Ossifying Fibroma
		Cementoblastoma
		Odontogenic Fibroma
		Odontogenic Myxoma
	Benign Mixed Epithelial and Mesenchymal Odontogenic Tumors	Ameloblastic Fibroma
		Dentinogenic Ghost Cell Tumor
		Odontoma
		Primordial Odontogenic Tumor
	Malignant Odontogenic Tumors	Ameloblastic Carcinoma
		Clear Cell Odontogenic Carcinoma
		Ghost Cell Odontogenic Carcinoma
		Odontogenic Carcinosarcoma
		Odontogenic Sarcoma
		Primary Intraosseous Carcinoma, NOS
		Sclerosing Odontogenic Carcinoma

**Table 2:** The 2022 WHO classification of odontogenic cysts of the jaws

Pathology	Cyst Subtype
Cysts of the Jaw	Calcifying Odontogenic Cyst
	Dentigerous Cysts
	Gingival Cysts
	Glandular Odontogenic Cyst
	Inflammatory Collateral Cysts
	Lateral Periodontal Cyst And Botryoid Odontogenic Cyst
	Nasopalatine Duct Cysts
	Odontogenic Keratocyst
	Orthokeratinised Odontogenic Cysts
	Radicular Cyst
	Surgical Ciliated Cysts

The data obtained was tabulated and imported into IBM SPSS Statistics for Windows, Version 29.0 (Released 2023; IBM Corp., Armonk, New York, United States). All descriptive statistics such as means, standard deviations, percentages, and frequencies were calculated and presented. The chi-square test was used to evaluate the associations between categorical variables, while continuum variables were analyzed using the one-way ANOVA test. Furthermore, the student t-test was also employed in the analysis of data such as differences between genders. A p-value of <0.05 was considered statistically significant.

## Results

A comprehensive analysis of patient records revealed that odontogenic cysts and tumors collectively accounted for 12.3% (n = 1889) of the overall processed specimens. Notably, within this subset, 53.7% (n = 1014) were identified as odontogenic tumors, while 46.3% (n = 875) manifested as odontogenic cysts. Statistical analysis utilizing a chi-square test of independence unveiled a gender-based discrepancy, with males exhibiting a higher prevalence than females. This discrepancy proved statistically significant (p < 0.001), establishing that men were markedly more susceptible to the diagnosis of odontogenic cysts and tumors in comparison to their female counterparts (Table 3).

**Table 3:** Sociodemographic characteristics of patients presenting with odontogenic cysts and tumors (N=1889) *Chi-square test assessing statistical association between gender distribution and pathology; **Student t-test assessing statistical differences in mean age at presentation between pathologies

Characteristic Pathology	Male, n (%)	Female, n (%)	p-value*	Total, n (%)	Age (years), mean ± SD	p-value**
Odontogenic cysts	634 (73.0)	241 (27.0)	< 0.001	875 (46.3)	26.47 ± 16.48	0.06
Odontogenic tumors	519 (51.2)	495 (48.8)		1014 (53.7)	32.80 ± 14.33	
Total	1153 (61.0)	736 (39.0)		1889	29.63 ± 15.41	

Both categories of pathologies manifested at comparable mean ages, with no discernible statistically significant differences. Stratifying the data into distinct age brackets revealed that the sum of both pathologies was most common within the age group of 21-30 years. Nonetheless, it was observed that odontogenic cysts predominated among individuals in the age group of 11-20 years, whereas tumors exhibited a higher prevalence within the age range of 21-30 years (Table 4). However, these findings, despite the observed patterns, did not demonstrate any statistically significant differences.

**Table 4:** Stratification of data into various age brackets (N=1889) *One-way ANOVA assessing the statistical difference between various age brackets of the pathologies

Age Group (years)	Odontogenic Cysts, n (%)	Odontogenic Tumors, n (%)	Total, n (%)	p-value*
0 – 10	68 (7.8)	36 (3.6)	104 (5.5)	0.6503
11 – 20	256 (29.3)	232 (22.9)	488 (25.8)	
21 – 30	213 (24.3)	309 (30.5)	522 (27.6)	
31 – 40	132 (15.1)	204 (20.1)	336 (17.8)	
41 – 50	72 (8.2)	98 (9.7)	170 (9.0)	
51 – 60	60 (6.9)	83 (8.2)	143 (7.6)	
61 – 70	49 (5.6)	34 (3.4)	83 (4.4)	
>70	25 (2.8)	18 (1.8)	43 (2.3)	

Odontogenic tumors

**Figure 2:**
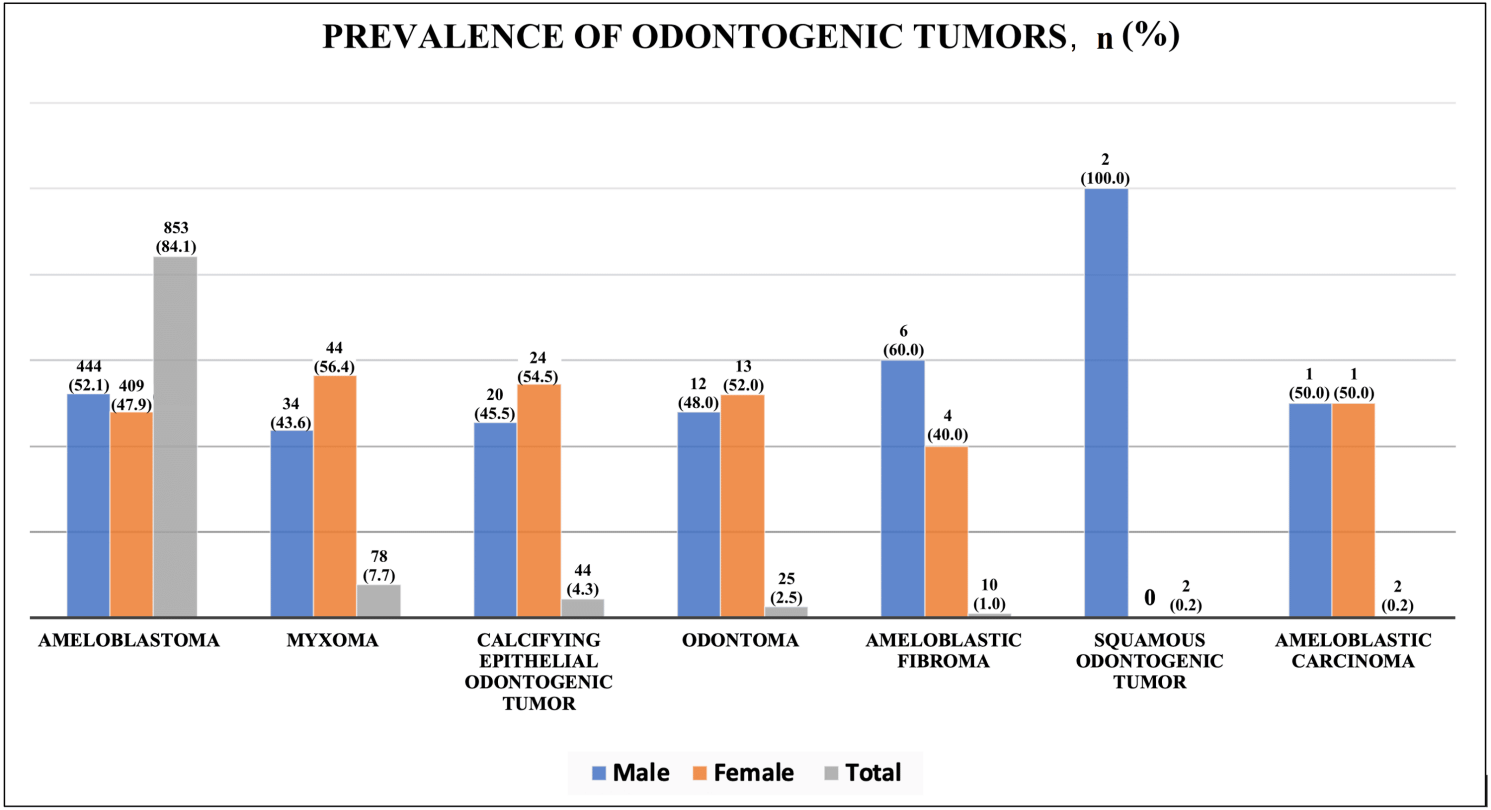
Bar chart showing the gender distribution of cases presenting with odontogenic tumors (N = 1014) Data are presented as n (%) and the total values (in gray) are calculated on the basis of the 1014 total odontogenic tumor cases reviewed

A total of 1014 odontogenic tumors were diagnosed during the study period. Ameloblastoma emerged as the predominant tumor, comprising 84.1% of cases. The distribution between males (52.1%) and females (47.9%) with ameloblastoma suggests a preponderance of this tumor in males. Among other significant tumor types, calcifying epithelial odontogenic tumors, myxoma, and odontoma also exhibited distinct gender-based prevalence. Calcifying epithelial odontogenic tumors and myxoma exhibited a higher proportion in females (54.5% and 56.4%, respectively). Odontoma exhibited a relatively even distribution between males and females. Ameloblastic fibroma, constituting 1.0% of cases, displayed a noteworthy gender disparity, with a 60% prevalence in males and 40% in females. The rare tumors, squamous odontogenic tumors and ameloblastic carcinoma, each contributed minimally (0.2% of cases). Squamous odontogenic tumors exclusively affected males (100%), while ameloblastic carcinoma presented an equal distribution between males and females (Figure 2).

The mean age was analyzed and assessed for various tumor diagnoses (Table 5). Odontoma emerged as the earliest diagnosed at a mean age of 19.28 ± 10.28 years, while ameloblastic carcinoma presented a notably delayed diagnosis at a mean age of 56.00 ± 36.77 years. Myxoma and ameloblastic fibroma displayed comparable ages at diagnosis of 26.43 ± 14.50 and 26.50 ± 7.17 years, respectively. Calcifying epithelial odontogenic tumor manifested at 28.82 ± 17.35 years, while ameloblastoma, the most prevalent benign lesion, exhibited diagnosis at 32.27 ± 15.34 years. Statistical analysis revealed a significant association between the total mean age of the patients and the type of tumor diagnosed (p-value < 0.001). When analyzed concerning gender, ameloblastoma, myxoma, and odontoma occurred earlier in males while calcifying epithelial odontogenic tumor, ameloblastic fibroma, and ameloblastic carcinoma occurred earlier among females. The only statistically significant finding between the gender and age at diagnosis was the myxoma (p-value <0.001). 

**Table 5:** Age distribution of cases presenting with odontogenic tumors (N=1014) *t-test assessing the differences of the mean age at presentation between gender; ** ANOVA assessing the statistical differences in mean age at presentation between various tumour groups.

Tumor Diagnosis	Age in Males (years), mean ± SD	Age in Females (years), mean ± SD	p-value*	Age in Overall Cases (years), mean ± SD	p-value**
*Benign Epithelial Odontogenic Tumors*					
Ameloblastoma	32.02 ± 15.40	32.54 ± 15.29	0.621	32.27 ± 15.34	< 0.001
Calcifying epithelial odontogenic tumor	31.20 ± 21.23	26.83 ± 13.47	0.432	28.82 ± 17.35	
Squamous odontogenic tumor	50.00 ± 45.26	-	-	50.00 ± 45.22	
Benign Mesenchymal Odontogenic Tumors					
Myxoma	18.60 ± 10.56	32.48 ± 14.32	<0.001	26.43 ± 14.50	
Benign Mixed Epithelial and Mesenchymal Odontogenic Tumors					
Odontoma	16.17 ± 8.56	22.15 ± 11.20	0.146	19.28 ± 10.28	
Ameloblastic fibroma	28.17 ± 8.13	24.00 ± 5.48	0.361	26.50 ± 7.17	
Malignant Odontogenic Tumors					
Ameloblastic carcinoma	82.00 ± 0.05	30.00 ± 0.05	-	56.00 ± 36.77	

Regarding the site of occurrence of odontogenic tumors, ameloblastoma exhibited a distinct predilection for the mandible, constituting 91.8% of cases. Conversely, only 4.9% of ameloblastoma were found in the maxilla, and 3.3% occurred in soft tissue. Calcifying epithelial odontogenic tumors demonstrates a similar pattern, with a notable prevalence in the mandible (61.4%) compared to the maxilla (38.6%). The squamous odontogenic tumor shows a predominance in the mandible. Myxoma and odontoma also display differential site predilections, with myxomas favoring the mandible (46.2%), while odontomas predominantly occur in the maxilla (60%). These findings suggest a varied propensity for different tumor types in specific anatomical sites within the oral cavity (Table 6). Ameloblastic carcinomas exhibit an equal distribution between the maxilla and mandible, each contributing 50%. The site of tumor and tumor diagnosis were found to display a significant association with one another (p-value <0.001); however, when scrutinized according to gender and site of tumor diagnosis, the results showed no association between the two.

**Table 6:** Site of presentation and gender distribution of odontogenic tumors (N=1014) *ANOVA assessing the statistical differences between the sites of presentation of various tumor groups

Tumor Diagnosis	Total Cases, n (%)	Primary Site									
		Maxilla			Mandible			Soft Tissue			
		Male, n	Female, n	Total, n (%)	Male, n	Female, n	Total, n (%)	Male, n	Female, n	Total, n (%)	p-value*
Benign Epithelial Odontogenic Tumors											
Ameloblastoma	853 (84.1)	23	19	42 (4.9)	407	376	783 (91.8)	14	14	28 (3.3)	<0.001
Calcifying epithelial odontogenic tumor	44 (4.3)	6	11	17 (38.6)	14	13	27 (61.4)	0	0	0 (0)	
Squamous odontogenic tumor	2 (0.2)	0	0	0 (0)	2	0	2 (100)	0	0	0 (0	
Benign Mesenchymal Odontogenic Tumors											
Myxoma	78 (7.7)	13	13	26 (33.3)	15	21	36 (46.2)	6	10	16 (20.5)	
Benign Mixed Epithelial and Mesenchymal Odontogenic Tumors											
Odontoma	25 (2.5)	8	7	15 (60)	4	5	9 (36)	0	1	1 (4)	
Ameloblastic fibroma	10 (1.0)	0	1	1 (10)	6	3	9 (90)	0	0	0	
Malignant Odontogenic Tumors											
Ameloblastic carcinoma	2 (0.2)	0	1	1 (50)	1	0	1 (50)	0	0	0	

Odontogenic cysts

**Figure 3:**
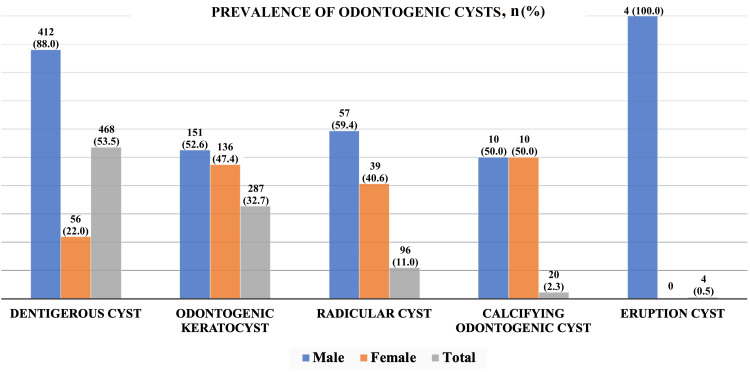
Bar graph showing the total number of cases and the gender distribution of odontogenic cysts (N = 875) Data are presented as n (%) and the total values (in gray) are calculated on the basis of the 875 total odontogenic cyst cases reviewed

A total of 875 odontogenic cysts were diagnosed during the study period. Dentigerous cysts were found to be most prevalent followed by the odontogenic keratocyst, radicular cysts, and calcifying odontogenic cysts while the eruption cyst was found to be most rare (Figure 3). In terms of gender differences, the dentigerous cysts exhibited a striking disparity, with a prevalence of 88.0% in males compared to 22.0% in females, collectively constituting 53.5% of all cases. Similarly, odontogenic keratocyst, radicular cyst, and the eruption cyst were all found to be more common among males while calcifying odontogenic cysts were found to be equal in both genders. 

Concerning the age at diagnosis, an interesting observation arises as the mean age for all cyst types falls within the fourth decade of life (30-40 years). Notably, the odontogenic keratocyst and dentigerous cyst exhibit an earlier onset, while the eruption cyst and calcifying odontogenic cyst manifests later within this age range (Table 7). After statistical analysis, no statistically significant correlation between the age at presentation and the type of cyst was found. In the context of gender, it was found that females diagnosed with odontogenic keratocysts, radicular cysts, calcifying odontogenic cysts, and eruption cysts tended to present at an earlier age than their male counterparts. Conversely, males with dentigerous cysts exhibited an earlier age at presentation compared to females. Remarkably, the divergent age at presentation between males and females with odontogenic keratocysts stands out as the sole statistically significant finding. 

**Table 7:** Age distribution of odontogenic cysts (N=875) *t-test assessing the differences of the mean age at presentation between gender; ** ANOVA assessing the statistical differences in mean age at presentation between various cyst groups.

Cyst Diagnosis	Age in Males (years), mean ± SD	Age in Females (years), mean ± SD	p-value*	Age in Overall Cases (years), mean ± SD	p-value**
Odontogenic keratocyst	30.99 ± 18.46	22.62 ± 14.88	<0.001	30.00 ± 18.26	0.894
Dentigerous cyst	29.54 ± 17.20	29.99 ± 17.02	0.82	30.00 ± 17.07	
Eruption cyst	37.75 ± 17.97	34.60 ± 12.91	0.07	36.00 ±17.97	
Calcifying odontogenic cyst	43.90 ± 25.70	31.70 ± 20.43	0.26	37.80 ± 23.45	
Radicular cyst	33.19 ± 18.14	27.00 ± 17.42	0.10	31.00 ± 18.02	

In terms of the site of presentation, odontogenic keratocysts, dentigerous cysts, and calcifying odontogenic cysts predominantly manifested in the mandible constituting 70.4%, 85.5%, and 55.0% of cases, respectively. Radicular cysts on the other hand exhibited a clear preference for the maxilla (53.1%). Statistical analysis revealed a significant correlation between the site of presentation and the cyst type (p<0.001); however, no significant correlation was found between the site and gender (Table 8).

**Table 8:** Site of presentation and gender distribution of odontogenic cysts (N=875) *ANOVA assessing the statistical differences between the site of presentation of various cyst groups

Cyst Diagnosis	Total cases, n (%)	Primary Site									
		Maxilla			Mandible			Soft tissue			
		Male, n	Female, n	Total, n (%)	Male, n	Female, n	Total, n (%)	Male, n	Female, n	Total, n (%)	p-value*
Odontogenic keratocyst	287 (32.7)	48	33	81 (28.2)	100	102	202 (70.4)	3	1	4 (1.4)	<0.001
Dentigerous cyst	468 (53.5)	48	20	68 (14.5)	364	36	400 (85.5)	0	0	0 (0.0)	
Eruption cyst	4 (0.5)	0	0	0 (0.0)	0	0	0 (0.0)	4	0	4 (100.0)	
Calcifying odontogenic cyst	20 (2.3)	4	4	8 (40.0)	6	5	11 (55.0)	0	1	1 (5.0)	
Radicular cyst	96 (11.0)	27	24	52 (53.1)	30	15	45 (46.9)	0	0	0 (0.0)	

## Discussion

This investigation offers a fresh perspective on the prevalence and distribution of odontogenic cysts and tumors in the Kenyan population, marking a significant update from prior reports almost a decade ago [17,18]. It is essential to emphasize that the distinctive variations observed across borders necessitate caution when extrapolating data from other population groups. Therefore, a meticulous examination of the clinicopathological characteristics of these lesions within the Kenyan population becomes paramount for advancing, understanding, and consequently refining patients’ treatment outcomes. Focused on the Nairobi Metropolitan area, the capital city of Kenya, this study draws from a population base that is reflective of the nation's demographic dynamics. The sample comprises referrals from neighboring counties, predominantly urban in nature, and emanates from two major referral hospitals in the country offering specialized oral and maxillofacial pathology services. Encompassing 25,032 samples derived from surgical biopsies spanning both pediatric and adult patient cohorts, this investigation offers a distinctive perspective on the frequencies and histological patterns diagnosed within the ambit of oral and maxillofacial pathology, diverging from previous reports and showcasing changing trends.

In the present study, tumors were a marginally frequent diagnostic group in comparison to cysts with a ratio of 1.2: 1 respectively. This marks a shift from the findings of a report by Dimba et al. in 2007, where the ratio of tumors to cysts was notably higher at 3.7:1 [16]. The current trend indicates an increase in cyst diagnoses over the intervening period. Also, this may potentially be influenced by the reclassification of odontogenic keratocyst as a cystic lesion instead of a tumor. There is a lack of regional African data on this matter, as the majority of studies focus on either cysts or tumors individually. Expanding beyond the African continent, an interesting reversal in the ratio of tumors to cysts is observed in the Portuguese population group, standing at 1:10.7 [26]. This pattern is also frequently documented among the Middle Eastern [27] and Australian [28] population groups. Genetic factors, environmental influences, lifestyle, and dietary habits may be some of the predominant factors giving rise to such discrepancies between population groups. In terms of age trends, the occurrence of cysts and tumors was observed at an average age of 26.47 ± 16.48 years and 32.80 ± 14.33 years, respectively. When comparing these findings to previous studies conducted on the Kenyan population group, the mean age of tumors aligns with the results reported in those studies [16]. However, interestingly the mean age for patients with cysts appears significantly higher than that reported previously by Butt et al. (25.05 years) [17] and Dimba et al. (23.7 years) [16], indicating a discrepancy in age trends for odontogenic cysts. Also, as stated previously, this may potentially be influenced by the reclassification of odontogenic keratocyst as a cystic lesion instead of a tumor. When compared to international data at large, the results of this study corroborate the findings in Nigeria [11], Libya [12], and Japan [29]. In contrast, European studies on the same indicate a higher mean age of both cysts and tumors with mean ages of 32.1 ± 18.1 and 37.1 ± 16.9 years, respectively [26].

In our study, the predominant odontogenic tumors were overwhelmingly benign (99.8%), with only a minimal occurrence of malignant tumors (0.2%). This distribution aligns with findings in both local and international reports [16]. Notably, ameloblastoma emerged as the most prevalent odontogenic tumor, accounting for a substantial 84.1%. Following this, myxoma represented 7.7% and calcifying epithelial odontogenic tumor constituted 4.3%. These results mirror those reported by Butt et al. [18] in 2012 among the Kenyan population and are consistent with studies conducted in China [30], Jamaica [31], Turkey [32], and India [33]. Conversely, regions such as the United States [34], Canada [35], Mexico [36], and Chile [37] exhibit odontoma as the most common odontogenic tumor. A review by Oladunni et al. in 2017 hypothesized that the surprisingly low prevalence of odontomas among Africans may be due to under-diagnosis of the lesion as a result of the lack of use of orthopantomograms and other diagnostic facilities [2]. However, after following the diagnostic protocol and extensive histological analysis of each specimen, this study maintains, in keeping with regional literature, that odontomas are relatively uncommon among Africans, indeed emphasizing the significant disparities in the prevalence of these lesions across borders and underscoring the need to generate regional data considering the uniqueness of each population.

It is important to acknowledge that although the odontogenic keratocyst has been frequently identified as the second most common tumor in various studies, its reclassification as a cyst precludes its inclusion in this specific category. These findings highlight the substantial impact of ameloblastoma on the local disease landscape, highlighting the urgency for resource allocation toward patient education and effective management of this particular tumor. Analyzing the site distribution of odontogenic tumors, our study unveiled a higher prevalence of lesions in the mandible than in the maxilla except for the odontoma, aligning with established literature [4,16,18]. A notable deviation from the existing body of research is the elevated incidence of soft tissue ameloblastomas and myxomas within our setting. Remarkably, scant literature addresses this, with most information confined to individual case reports [38]. Larger scale studies have thus far failed to document occurrences of this specific tumor site.

The examination of specimens in the current study revealed a prevalence of odontogenic cysts of 46.3%. This is markedly higher than the 14.41% reported by Dimba et al. in 2007 [16] and the subsequent decrease to 4.56% reported by Butt et al. in 2011 [17]. The decline in 2011 may be attributed to the reclassification of the odontogenic keratocyst as a benign neoplasm rather than an odontogenic cyst. In this investigation, the dentigerous cyst emerged as the most common (53.5%), followed by odontogenic keratocyst (32.7%) and the radicular cyst (11.0%). The calcifying odontogenic cysts and eruption cysts were observed in only 2.3% and 0.5% of cases, respectively. Males exhibited a higher frequency compared to females, with a ratio of 2.6:1. These findings deviate from prior observations in the Kenyan population, where the odontogenic keratocyst previously ranked as the most prevalent, followed by the dentigerous and radicular cysts, respectively [16]. Butt et al.'s 2011 update indicated a shift, with the dentigerous cyst being the most common, followed by the radicular cyst due to the reclassification of the keratocyst as an odontogenic tumor during this time [17]. However, despite its current reclassification as a cyst according to the 2022 WHO classification [20], it becomes evident that the dentigerous cyst may have indeed become more prevalent than the keratocyst in this region. Nevertheless, the underlying reason for this shift remains unknown. In contrast to international literature, the observed ratio of males to females affected exceeds that reported for the Libyan and Indian populations [12,33]. Additionally, the average age of presentation among the Libyans and British population groups exceeded 40 years, while in the current study, it was 30 years [12,39]. The prevailing cyst type was the radicular cyst, noted in the Canadian [35], Mexican [40], Libyan [12], and Pakistani groups [41].

The present study provided an update on the prevalence of both cysts and tumors within the Kenyan population. In contrast to findings in Western Africa, there was scant evidence of a transition from cysts to tumors in this population group, in fact, a rise in cyst numbers was observed when compared to previous studies [24]. Although the burden of odontogenic carcinomas such as ameloblastic carcinoma was minimal in this study, the late presentation of patients with advanced tumors continues to present challenges in diagnosis and patient management. In light of the above findings, it would be prudent to establish a comprehensive and centralized database of clinicopathological characteristics of odontogenic cysts and tumors incorporating a wider range of demographic and clinical information of each patient, allowing a more accurate understanding of the disease landscape in Kenya. There should be a concerted effort to raise awareness among healthcare professionals emphasizing the unique variations observed within this population and avoiding extrapolation of data from other population groups.

Limitations

Some limitations of this epidemiological analysis are that it did not investigate the underlying causes for the observed changes in the trends of odontogenic cysts and tumors in Kenya. Additionally, it would have been beneficial to collect data from more centers to obtain a larger sample size that is more representative of the population. Finally, the retrospective design of the study may have introduced bias during the collection of data. Nevertheless, the study demonstrates significant changes observed from previous reports within the Kenyan population which necessitate the reallocation of resources to improve the diagnosis and management of the prevalent cyst and tumor types. The trends related to odontogenic cysts and tumors have indeed changed in Kenya. 

## Conclusions

This study provides a contemporary view of odontogenic cysts and tumors in Kenya. Notable shifts in the prevalence of cysts and tumors, particularly the rise in cyst cases, highlight the dynamic nature of these conditions. The predominance of benign tumors, with ameloblastoma as the most prevalent, underscores the need for targeted resource allocation in patient education and management. The study also reveals unique aspects, such as the incidence of soft tissue ameloblastomas and myxomas, emphasizing the importance of region-specific research. To improve diagnosis and management, establishing a comprehensive database, raising awareness among healthcare professionals, and reallocating resources to address the evolving disease landscape are recommended.

## References

[1] Kaimenyi JT (2004). Oral health in Kenya. Int Dent J.

[2] Ogundana OM, Effiom OA, Odukoya O (2017). Pattern of distribution of odontogenic tumours in sub-Saharan Africa. Int Dent J.

[3] Oginni FO, Stoelinga PJ, Ajike SA (2015). A prospective epidemiological study on odontogenic tumours in a black African population, with emphasis on the relative frequency of ameloblastoma. Int J Oral Maxillofac Surg.

[4] Aregbesola B, Soyele O, Effiom O, Gbotolorun O, Taiwo O, Amole I (2018). Odontogenic tumours in Nigeria: a multicentre study of 582 cases and review of the literature. Med Oral Patol Oral Cir Bucal.

[5] Parkins GE, Armah GA, Tettey Y (2009). Orofacial tumours and tumour-like lesions in Ghana: a 6-year prospective study. Br J Oral Maxillofac Surg.

[6] Mamabolo M, Noffke C, Raubenheimer E (2011). Odontogenic tumours manifesting in the first two decades of life in a rural African population sample: a 26 year retrospective analysis. Dentomaxillofac Radiol.

[7] Neway M, Eshete S, Minasse M (1994). Oro-facial tumours in Ethiopian patients. J Cranio-Maxillofac Surg.

[8] Chidzonga MM, Lopez VM, Alverez AP (1996). Odontogenic tumours: analysis of 148 cases in Zimbabwe. Cent Afr J Med.

[9] Abdennour S, Benhalima H (2013). Benign odontogenic tumours: epidemiological analysis of 97 cases in the Algerian population [Article in French]. Rev Stomatol Chir Maxillofac Chir Orale.

[10] Al-Aroomy L, Wali M, Alwadeai M, Desouky EE, Amer H (2022). Odontogenic tumors: a retrospective study in Egyptian population using WHO 2017 classification. Med Oral Patol Oral Cir Bucal.

[11] Lawal A, Adisa A, Sigbeku O (2012). Cysts of the oro-facial region: a Nigerian experience. J Oral Maxillofac Pathol.

[12] Buaoud MM, Musrati A, Hagstrom J (2023). Prevalence of odontogenic cysts in a group of Libyan population: a retrospective study. Niger J Clin Pract.

[13] Mohammed M, Mahomed F, Ngwenya S (2019). A survey of pathology specimens associated with impacted teeth over a 21-year period. Med Oral Patol Oral Cir Bucal.

[14] Cameron HM (1973). Oral tumours in Kenya. Pathol Microbiol (Basel).

[15] Wakoli R, Bhaji A (1987). A retrospective study of jaw cysts in a Kenyan Hospital. Afr Dent J.

[16] Dimba EA, Gichana J, Limo AK, Wakoli KA, Chindia ML, Awange DO (2007). An audit of oral diseases at a Nairobi centre, 2000-2004. Int Dent J.

[17] Butt FM, Ogeng'o J, Bahra J, Chindia ML (2011). Pattern of odontogenic and nonodontogenic cysts. J Craniofac Surg.

[18] Butt FM, Guthua SW, Awange DA, Dimba EA, Macigo FG (2012). The pattern and occurrence of ameloblastoma in adolescents treated at a university teaching hospital, in Kenya: a 13-year study. J Craniomaxillofac Surg.

[19] Franklin JR, Vieira EL, Brito LN, Castro JF, Godoy GP (2021). Epidemiological evaluation of jaw cysts according to the new WHO classification: a 30-year retrospective analysis. Braz Oral Res.

[20] Soluk-Tekkesin M, Wright JM (2022). The World Health Organization Classification of Odontogenic Lesions: a summary of the changes of the 2022 (5th) edition. Turk Patoloji Derg.

[21] (2022). World Health Organization: WHO classification of tumors online. https://tumourclassification.iarc.who.int/welcome/.

[22] Iyogun C, Ochicha O, Sule A (2013). Jaw cysts in Kano: northern Nigeria. Int J Oral Maxillofac Pathol.

[23] Akinmoladun V, Udeabor S, Arotiba J (2011). Pattern of odontogenic tumours in Nigeria: a review of the literature. Niger Dent J.

[24] Omoregie FO, Sede MA, Ojo AM (2015). Ameloblastomatous change in radicular cyst of the jaw in a Nigerian population. Ghana Med J.

[25] Onyango JF, Omondi BI, Njiru A, Awange OO (2004). Oral cancer at Kenyatta National Hospital, Nairobi. East Afr Med J.

[26] Monteiro L, Santiago C, Amaral BD, Al-Mossallami A, Albuquerque R, Lopes C (2021). An observational retrospective study of odontogenic cyst´s and tumours over an 18-year period in a Portuguese population according to the new WHO head and neck tumour classification. Med Oral Patol Oral Cir Bucal.

[27] Baghaei F, Zargaran M, Najmi H, Moghimbeigi A (2014). A clinicopathological study of odontogenic cysts and tumors in Hamadan, Iran. J Dent (Shiraz).

[28] Kelloway E, Ha WN, Dost F, Farah CS (2014). A retrospective analysis of oral and maxillofacial pathology in an Australian adult population. Aust Dent J.

[29] Kokubun K, Yamamoto K, Nakajima K (2022). Frequency of odontogenic tumors: a single center study of 1089 cases in Japan and literature review. Head Neck Pathol.

[30] Wu PC, Chan KW (1985). A survey of tumours of the jawbones in Hong Kong Chinese: 1963-1982. Br J Oral Maxillofac Surg.

[31] Ogunsalu CO (2003). Odontogenic tumours from two centres in Jamaica. A 15-year review. West Indian Med J.

[32] Soluk-Tekkesin M, Cakarer S, Aksakalli N, Alatli C, Olgac V (2020). New World Health Organization classification of odontogenic tumours: impact on the prevalence of odontogenic tumours and analysis of 1231 cases from Turkey. Br J Oral Maxillofac Surg.

[33] Nalabolu GR, Mohiddin A, Hiremath SK, Manyam R, Bharath TS, Raju PR (2017). Epidemiological study of odontogenic tumours: an institutional experience. J Infect Public Health.

[34] Buchner A, Merrell PW, Carpenter WM (2006). Relative frequency of central odontogenic tumors: a study of 1,088 cases from Northern California and comparison to studies from other parts of the world. J Oral Maxillofac Surg.

[35] Daley TD, Wysocki GP, Pringle GA (1994). Relative incidence of odontogenic tumors and oral and jaw cysts in a Canadian population. Oral Surg Oral Med Oral Pathol.

[36] Gaitán-Cepeda LA, Quezada-Rivera D, Tenorio-Rocha F, Leyva-Huerta ER (2010). Reclassification of odontogenic keratocyst as tumour. Impact on the odontogenic tumours prevalence. Oral Dis.

[37] Mosqueda-Taylor A, Irigoyen-Camacho ME, Diaz-Franco MA, Torres-Tejero MA (2002). Odontogenic cysts. Analysis of 856 cases [Article in English, Spanish]. Med Oral.

[38] Suma MS, Sundaresh KJ, Shruthy R, Mallikarjuna R (2013). Ameloblastoma: an aggressive lesion of the mandible. BMJ Case Rep.

[39] Jones AV, Craig GT, Franklin CD (2006). Range and demographics of odontogenic cysts diagnosed in a UK population over a 30-year period. J Oral Pathol Med.

[40] Ledesma-Montes C, Hernández-Guerrero JC, Garcés-Ortí M (2000). Clinico-pathologic study of odontogenic cysts in a Mexican sample population. Arch Med Res.

[41] Akram S, Naghma Naghma, Anwar M, Shakir MM (2013). Prevalence of odontogenic cysts and tumors in Karachi, Pakistan. J Dow Univ Health Sci.

